# Vasoreactive Pulmonary Arterial Hypertension Manifesting With Misleading Epileptic Seizure: Diagnostic and Treatment Pitfalls

**DOI:** 10.3389/fped.2019.00262

**Published:** 2019-07-04

**Authors:** Nesrine Farhat, Bjorn Cools, Marc Gewillig, Marie-Christine Seghaye, Yacine Aggoun, Maurice Beghetti

**Affiliations:** ^1^Department of Pediatrics, University Hospital Liège, Liège, Belgium; ^2^Pediatric Cardiology Unit and Centre Universitaire Romand de Cardiologie et Chirurgie Cardiaque Pédiatrique, Lausanne University Hospital (CHUV), Geneva University Hospitals (HUG), University of Geneva, Geneva, Switzerland; ^3^Pediatric Cardiology Unit and Centre Universitaire Romand de Cardiologie et Chirurgie Cardiaque Pédiatrique, Lausanne University Hospital (CHUV), Geneva University Hospitals (HUG), University of Lausanne, Lausanne, Switzerland; ^4^Department of Pediatric Cardiology, University Hospital Leuven, Leuven, Belgium

**Keywords:** pediatric, calcium channel blockers, vasoreactivity testing, syncope, pulmonary arterial hypertension

## Abstract

A 5-year-old girl presented with acute nocturnal episodes of loss of consciousness following abdominal pain and crying. Epilepsy was primarily diagnosed but the course of the disease was suggestive of pulmonary hypertension. An adapted invasive assessment of pulmonary pressure and pharmacological challenge allowed for diagnosing vasoreactive pulmonary arterial hypertension. Initial treatment with sildenafil was not effective. Thus, calcium channel blockers were introduced when positive vasoreactivity was confirmed and permitted to stop the occurrence of the syncope and dramatically improved clinical status. At 2 years follow-up she is well without any complaint and in functional class I. Echocardiography shows a slightly enlarged but not hypertrophied right ventricle with a nearly normalized estimated right ventricular pressure. The last catheterization shows subnormal values of pulmonary arterial pressure (mean pulmonary artery pressure: 24 mmHg) and pulmonary vascular resistance (5, 4 Wood units^*^m^2^), normalizing with inhaled Nitric Oxide (mean pulmonary artery pressure of 14 mmHg and pulmonary vascular resistance of 1.5 Wood units^*^m^2^). Vasoreactive pulmonary arterial hypertension is a rare entity in children but it should not be misdiagnosed with seizures due to the presence of syncopal episodes. According to current knowledge, this form seems to have a better prognosis than non-reactive pulmonary arterial hypertension and the treatment of choice remains as calcium channel blockers. The management of this case was characterized by successive mishaps and potentially harmful mistakes and underscores the potential risk with pediatric PH evaluation in non-expert centers.

Pulmonary arterial hypertension (PAH) in children is a rare and severe disorder with significant morbidity and mortality (1–6). Despite improvement of its prognosis there is still dismal data about the longterm outcomes in children (1, 4, 7, 8). The management of pediatric PAH is still partially based on the adult strategy validated by many pediatric recommendations rather than controlled studies (1, 3, 4, 7–9). However, international experts tried to find consensus and give recommendations to best manage children with PAH (1, 3). According to the last guidelines of European Society of Cardiology and European Respiratory Society (ESC/ERS) PAH can be defined as a mean pulmonary artery pressure (mPAP) higher than 25 mmHg with a normal pulmonary capillary wedge pressure (PCWP) of ≤15 mmHg and increased pulmonary vascular resistances index (PVRI) >3 Wood units^*^m^2^ (3, 4, 8–11). In addition, values between 20 and 24 mmHg cannot be considered normal and this may be reconsidered following the last world symposium on pulmonary hypertension (12). However, this definition may be not easily applicable to all children but has been accepted in recent pediatric recommendations (1, 3). The most frequent etiologies of PAH in the pediatric population, is idiopathic (IPAH), heritable (HPAH) or associated with congenital heart disease (4, 7, 8). IPAH or HPAH represents 57% of all PAH registered by the TOPP (Tracking outcomes and Practice in Pediatric Pulmonary Hypertension) (1, 7, 9, 10). The estimation of the incidence for IPAH varies from 0.48 to 0.7 per million children and the prevalence from 2.1 to 4.4 per million, depending on the country where the studies were conducted (1).

Vasoreactive PAH is a particular form of PAH, the definition of which was not based on a clear consensus in children (1, 5, 6, 13–15). Vasoreactivity is defined by the presence of a positive response to vasodilators at invasive pharmacological challenge (4, 8). The REVEAL-pediatric criteria (modified of BARST criteria) are most often used in children that request a decrease of at least 20% of the mPAP with unchanged, increased or <10% decreased CI, and unchanged or decrease pulmonary to systemic vascular resistance ratio (4, 6, 13, 14, 16). Recently Douwes et al. reported that the Sitbon criteria (reduction of 10% in the mPAP, reach an absolute value less or equal than 40 mmHg and unchanged or increased cardiac output) are the most adapted to determine the vasoreactivity in children and are the most adapted and selective for children who will have the best outcome with CCB treatment (5, 15). Using these criteria allows to observe the same incidence of vasodilator responders in children and adults, varying from 5 to 17% (5). Earlier, when REVEAL criteria were used, the incidence of vasodilator responders in children was estimated to be 10 to 40%, depending on the studies (1, 8, 14, 17–19).

Even if not completely elucidated, pathophysiology of this disease seems to be consecutive to intracellular pathway dysfunction resulting in a shift in the vasoactive balance toward an exaggerated vasoconstrictory response (8–11, 20). In this particular form of reactive PAH, clinical manifestations may be related to acute pulmonary hypertensive crises triggered by several types of stimuli. Unlike other forms of PAH where the clinical manifestations are mainly due to RV failure and decreased of exercise capacity. These crises may result in acute life threatening decrease of cardiac output and syncope. The paroxysmal nature of vasoreactive PAH crisis renders the diagnosis challenging. For that reason, diagnosis needs invasive hemodynamic investigation with acute vasoreactivy testing. Recently it was suggested at the last PH world symposium to create a specific subgroup of vasoreactive PAH in the classification. It is of outmost importance to discriminate the vasoreactive responders because calcium channel blockers (CCB) therapy are correlated with an excellent prognosis (1, 3–6).

We describe a 5-year-old girl with a presentation of vasoreactive PAH. She presented with nocturnal paroxysmal episodes of loss of consciousness which have first been mistaken for epileptic seizure.

## Case Report

A 5-year-old girl without any contributively family or personal history was admitted at night to the emergency department because of an episode of loss of consciousness of a few seconds that followed abdominal pain, crying and urges. The parents observed pallor, cyanosis, loss of muscular tone and ocular revulsion. For a few months, she frequently complained about abdominal pain with short episodes of absence. Parents also reported a frequent cough and a reduction of exercise tolerance.

At admission, physical examination shows a systolic regurgitant murmur. Laboratory investigations showed a slight metabolic acidosis, increased concentrations of ultrasensitive Troponin T (65 ng/l) and of liver transaminases (ASAT 82U/l and ALAT 48U/l). N-terminal pro-Brain Natriuretic Peptide (NT-Pro-BNP) levels were elevated at 1,024 ng/l (normal value 12–214 ng/l).

Electrocardiogram (ECG) showed an incomplete right bundle branch block, compatible with a right ventricular hypertrophy. Echocardiography showed a slight enlargement of the right ventricle (RV) and right atrium, a tricuspid regurgitation that allowed the estimation of elevated systolic right ventricular pressure of more than 45 mmHg and pulmonary artery dilatation. Putative diagnosis in the emergency department was a severe seizure equivalent with secondary pulmonary hypertension and myocardial cell damage due to hypoxemia and acidosis.

During the next hours, the patient collapsed three times again since she was monitored. The crisis started with a tachycardia associated with a decrease of oxygen saturation followed by bradycardia and tonico-clonical seizures. The patient recovered spontaneously following the first crisis. Intrarectal diazepam was given to successfully stop the two other crises. Electro-encephalography (EEG) was compatible with the presence of partial epileptic seizures. Cerebral magnetic resonance imaging was normal. A treatment with valproic acid was started. Echocardiography was controlled on the next day that confirmed dilatation of the right cardiac cavities, a tricuspid regurgitation III/IV and an estimated RV pressure of 55 mmHg (half-systemic) suggesting the presence of pulmonary hypertension. Sleep apnea syndrome was excluded by polysomnography. Laboratory investigations fully normalized at day 2. After improvement under anti-comitial therapy she was discharged with the putative diagnosis of frequent partial epileptic seizures responsible for prolonged hypoxia and pulmonary hypertension.

However, she became increasingly breathless when exercising, especially at late afternoon and in the evening. She continuously complained about abdominal pain. Subsequent echocardiography showed a dilatation of the right atrium and right ventricle with an estimated RV pressure of 75–80 mmHg. There was sign of left ventricular compression with a flattening aspect of the interventricular septum.

Two weeks after the hospital discharge, she was therefore referred for invasive hemodynamic exploration performed under general anesthesia. Pulmonary artery pressure was 33 mmHg for the systolic, 17 mmHg for the diastolic and 25 mmHg for the mean pulmonary artery pressure (mPAP) and pulmonary vascular resistances index (PVRI) were elevated at 4.5 Wood units ^*^m^2^, reaching 58% of systemic values. There was no fall of pulmonary arterial pressure with 100% of oxygen (O_2_). Vasoreactivity testing with inhaled nitric oxide (iNO) was not performed at that time and treatment with sildenafil was started at a dose of 10 mg 3 times daily (1.5 mg/kg/d). The valproic acid was switched to levetiracetam. Complete and exhaustive investigations following current recommendations were performed to exclude secondary PAH. During hospital stay, despite treatment with sildenafil and levetiracetam, she had another episode with tachycardia, loss of consciousness leading to respiratory arrest and hypotension justifying a transfer to the intensive care unit. The EEG registered during this incident showed a lack of electrical activity due to a low cardiac- and cerebral output. Patient underwent successful resuscitation.

A second invasive hemodynamic study was performed 5 days later with vasoreactivity testing with iNO showing a significant decrease of pulmonary arterial pressure and resistances (Table 1). The mPAP decreased from 31 to 17 mmHg with combination of O_2_ and iNO. The CI stayed above 3.5 L/min/m^2^ during vasoreactivity testing and the PVRI fell from 5.4 to 0.7 Wood units ^*^m^2^. Subsequent to the catheterization she was transferred to the intensive care unit (ICU) with a Swan-Ganz catheter in place. On ICU she developed another paroxysmal episode initiated with sinus tachycardia 170 BPM with acute increase of the systolic PAP of 70 mmHg. Simultaneous echocardiography showed acute dilation of the right ventricle (RV) with important D-shape of the interventricular septum (IVS) and increased systolic RV pressure measured of tricuspid regurgitation. The episode resolved by administration of iNO through nasal cannula.

**Table 1:** Cardiac catheterization with vasoreactivity testing.

	**Cardiac catheterization under sildenafil treatment**	
	**Baseline**	**O_**2**_ 100%+NO 20 ppm**
PAP(s/d-m) mmHg	45/20/31	25/12/17
Ao(s/d-m) mmHg	83/45/62	76/45/58
PCWP mmHg	12	13
CO L/min	2.5	4
CI L/min/m^2^	3.5	5.5
PVRI WUm^2^	5.4	0.7
PAPm/SAPm	0.5	0.29

*PAP, pulmonary artery pressure; Ao, aorta; O_2_, oxygen; NO, inhaled nitric oxide; s/d-m, systolic/diastolic and mean; PCWP, pulmonary capillary wedge pressure; CI, cardiac index; PVRI, pulmonary vascular resistance index; PAPm, mean pulmonary artery pressure; SAPm, mean systemic artery pressure*.

Calcium antagonist diltiazem was added to the treatment. Initially, there were still some events but progressively less important and no additional need for iNO administration. The epileptic treatment was stopped. After a couple of days, she could be discharged with no more recurrence of syncope. Diagnosis of vasoreactive PAH with low cardiac output, hypoxia and consecutive epileptic seizure was made. The vasodilator treatment was gradually increased to achieve high doses of diltiazem (12 mg/kg/d) and sildenafil (3 mg/kg/d).

Two and half years later, the clinical evolution is satisfactory with no single acute incidents reported and no complaints. The exercise tolerance is good without any limitation of physical activity (functional class I). The controlled cardiac MRI showed a normal function of the right ventricle without sign of dilatation. Laboratory investigations showed normal concentrations of ultrasensitive Troponin T (<3 ng/l) and NT-Pro-BNP (78 ng/l).

The pulmonary arterial pressure measured during last cardiac catheterization was 36/14/24 mmHg and decreased to 19/12/14 under iNO (20 ppm) and O_2_. PVRI fell from 5.4 to 1.5 Wood units ^*^m^2^. The last 6 min walking test showed a walking distance of 470 m without desaturation, considered lower limit for the age (21). Echocardiography currently shows slight dilation of the RV with minimal tricuspid regurgitation allowing to measure a systolic RV pressure of 28 mmHg and good RV function.

## Discussion

PAH is a rare but severe disease. Without adequate management, prognosis remains poor, leading to right ventricular dysfunction and death (7–9, 11, 13, 16–18, 22). In children, the most frequent manifestation of PAH is dyspnea on exertion, fatigue and often syncope during or subsequent to exercise. Less frequent but not rare symptoms are seizures, chest pain and signs of right heart failure (2, 8–10, 20). In infant evocative signs of PAH are tachycardia, loss of appetite, growth retardation and even lethargy (11).

Our patient shows the particular presentation of nocturnal paroxysmal convulsive seizures as presenting a form of PAH which was first mistaken for epileptic seizures. Among the differential diagnosis of syncope in children, a cardiopulmonary origin is rare but, as illustrated by our case, it can be the first sign of PAH. In contrast to adults, children with PAH show syncope as often as in about 20% of cases (2). In adults with PAH, the presence of syncope persisting despite treatment is associated with a higher risk of sudden death. In children with PAH, the prognostic significance of syncope has still to be established by longitudinal follow-up studies (2).

The gold standard for the diagnosis of PAH remains the invasive measurement of hemodynamics with acute vasoreactivity testing (AVT). This testing is central to determine treatment strategy (1, 3, 4, 6, 8, 10, 11, 14, 15). The cardiac catheterization should be done by an experienced pediatric team to minimize morbidity (1, 3, 5, 6). The TOPP registry reported a risk of approximately 5.9% of complications. These complications include pulmonary hypertensive crises, cardiac arrests, need for inotropic support, arrhythmias and pulmonary hemorrhage (6, 7).

Recent recommendations underline the importance of vasoreactivity testing and agree for the treatment by CCB in case of positive testing. This leads to improvement of WHO functional class and long term survival in children (1, 5, 7, 8, 10, 11, 14, 19). In line with this, initiation of CCB, in our case showed dramatic clinical improvement without any recurrence of syncope or complaints and normalized hemodynamics at cardiac catheterization. In our case, vasoreactivity testing was not performed at first hemodynamic evaluation and thus full diagnosed was not obtained. Once the diagnosis was established the use of CCB lead to significant clinical improvement and near normalization of pulmonary pressures. Vasoreactive patients should be treated with CCB and there are no data supporting the use of other vasodilators in this specific subgroup of patients. The role of acute vasodilatory testing was also described in an adult patient with severe PAH presenting as recurrent syncope and severe hemodynamics comprise (23).

In summary, because syncope is a frequently presenting symptom of pediatric PAH, it should be considered in the differential diagnosis of syncopal episode. Vasoreactive PAH must be identified as early as possible to be adequately managed. Thus, vasoreactivity testing is essential and must be performed in order to guide treatment. CCB remains the treatment of choice for vasoreactive responders allowing for excellent outcomes compared to other forms of PAH. The management of this case was characterized by successive mishaps and potentially harmful mistakes and illustrates that pediatric PAH should be diagnosed and followed up in expert centers.

## Data Availability

No datasets were generated or analyzed for this study.

## Ethics Statement

The parents gave written informed consent for the publication for their daughter's case report.

## Author Contributions

All the authors participated in the care of the patient. NF and MB have written this case report which has been validated by all the authors.

### Conflict of Interest Statement

MB reports non-financial support, grants and personal fees from Actelion Pharmaceuticals Ltd; grants and personal fees from Bayer HealthCare; and consultancy fees from Eli Lilly, Pfizer and GSK. BC consults for Actelion Pharmaceuticals Ltd and Bayer HealthCare and has received a research grant from Bayer HealthCare. The remaining authors declare that the research was conducted in the absence of any commercial or financial relationships that could be construed as a potential conflict of interest.
